# Dynamics of adolescents’ smartphone use and well-being are positive but ephemeral

**DOI:** 10.1038/s41598-022-05291-y

**Published:** 2022-01-25

**Authors:** Laura Marciano, Charles C. Driver, Peter J. Schulz, Anne-Linda Camerini

**Affiliations:** 1 Institute of Public Health, USI Università della Svizzera Italiana, Via Buffi 13, 6900 Lugano, Switzerland; 2 Institute of Education, University of Zurich, Freiestrasse 36, 8032 Zurich, Switzerland; 3 Faculty of Communication, Culture, and Society, USI Università della Svizzera Italiana, Via Giuseppe Buffi 13, 6900 Lugano, Switzerland

**Keywords:** Psychology, Human behaviour

## Abstract

Well-being and smartphone use are thought to influence each other. However, previous studies mainly focused on one direction (looking at the effects of smartphone use on well-being) and considered between-person effects, with self-reported measures of smartphone use. By using 2548 assessments of well-being and trace data of smartphone use collected for 45 consecutive days in 82 adolescent participants (M_age_ = 13.47, SD_age_ = 1.62, 54% females), the present study disentangled the reciprocal and individual dynamics of well-being and smartphone use. Hierarchical Bayesian Continuous Time Dynamic Models were used to estimate how a change in frequency and duration of smartphone use predicted a later change in well-being, and vice versa. Results revealed that (i) when participants used the smartphone frequently and for a longer period, they also reported higher levels of well-being; (ii) well-being positively predicted subsequent duration of smartphone use; (iii) usage patterns and system dynamics showed heterogeneity, with many subjects showing reciprocal effects close to zero; finally, (iv) changes in well-being tend to persist longer than changes in the frequency and duration of smartphone use.

## Introduction

Compared to any other digital media device, the smartphone is experienced as a “cognitive attractor”^1^ since it delivers short moments of satisfaction at a low cost and in combination with a high salience of the stimulus. Real-time data^2^ showed that smartphone use is a deeply internalized and reinforced behaviour, with the potential of disrupting the flow of other ongoing activities. Due to its pervasiveness, public concern about the effect of smartphones on youth well-being has been raised^3–5^, especially considering that depressive symptoms^6^ grew after the introduction of digital technologies^7,8^. Although the two trends have changed together, this does not necessarily imply that one causes the other. Instead, (lower) well-being may also enhance digital media use^9,10^. In particular, by looking at the effects of depressive symptoms and self-reported screen-based activities, a previous study found positive but small effects and different trajectories linking the two concepts^11^. Other studies modelled between-versus within-person effects^12–15^, leading to mixed results. Yet, a meta-analysis looked at trace data of digital media use and found that self-reports seldom accurately estimated trace data^16^. There are three main shortcomings of previous studies: (i) They mainly looked at one direction of the effects only (i.e., how media use influences well-being), (ii) although some studies considered within-person effects, they mainly modelled between-person associations, and (iii) they employed self-reported measures of smartphone use. To overcome these limitations at once, the present study investigated how well-being and smartphone use (in terms of frequency and duration of use) influence each other over time in a sample of adolescent participants. We relied on intensive longitudinal data, i.e. in-situ assessments of well-being and trace data of smartphone use collected for 45 consecutive days, to model the day-by-day, reciprocal, and individual dynamics of smartphone use and well-being. Using self-report and objectively trace data, combined with an advanced statistical approach, i.e. Hierarchical Bayesian Continuous Time Dynamic Modeling, allowed us to disentangle person-specific dynamics over time and to make predictions on present and future states. By doing so, we tackled simultaneously in one exploratory study all three limitations (the focus on the effects from smartphone use to well-being mostly, between-persons design mostly, and self-reported measures), with the hope to reconcile contradictory findings in prior research.

### Reciprocal effects in media research

The evaluation of well-being in relation to digital media use has become crucial considering that mental health problems^17–19^ frequently start in adolescence, which is also the age of heaviest smartphone use^20^. Although well-being goes beyond the absence of mental health problems^21^, previous literature mainly focused on the link between the latter (in terms of depressive symptoms, anxiety, loneliness, poor sleep outcomes, stress, hyperactivity, and health-related problems, to cite some) and digital media use in youth, finding positive relationships^22–29^. At least four reviews of reviews^30–33^ summarized the existing literature on this link, and another one^21^ summed up previous findings also conceptually and meta-analytically. They all led to mixed conclusions and pointed towards small effects in both negative and positive directions. The reviews mainly included cross-sectional findings on between-person (i.e., trait-like) associations, limiting interpretations of how changes arise^25^. Although a recent narrative review summarized results of fourteen longitudinal studies on well-being and digital media use in adolescence^34^, the authors also reported contradicting effects. Further, they suggested that longitudinal effects may be bidirectional. To overcome limitations of past studies and shed light on the mixed findings, researchers started to use statistical models to study within-person effects^35–37^ in longitudinal data.

Using large-scale representative panel data, Orben and colleagues^15^ modelled both between-person associations and within-person effects among adolescents’ social media use and life satisfaction. They found that social media effects were “nuanced, small at best, reciprocal over time (…) and contingent on analytic methods”^15^^(p. 10226)^. Considering factors like the family context and digital media device ownership, another study found a positive, small, and bidirectional within-person effect between Internet use and depression in 981 early adolescents followed over 4 years^38^. However, other studies did not find cross-lagged relationships when controlling for between-person associations. For example, following 500 adolescents over 8 years, Coyne et al.^39^ reported that time spent on social media was moderately related to anxiety and depression at the between-person level, thus corroborating many cross-sectional studies finding negative associations. However, within-person-effects analyses did not reveal any temporal associations between the two variables. The intensity of social media use and mental health did not predict each other at the within-person level in another study with 2109 early adolescents followed over 2 years by Boer et al.^40^. Furthermore, Jensen et al.^41^ collected Ecological Momentary Assessments (EMAs) data in 388 adolescents over the course of 2 weeks, focusing on internalizing and externalizing symptoms. They found that daily technology use, including social media and smartphone use for different purposes, was not associated with mental health symptoms. The data only supported limited daily quadratic associations, i.e. adolescents reported lower mental health on days they did not use the smartphone at all or when they used it excessively. Other investigators described that well-being might influence digital media but not the opposite. For example, lower well-being predicted small increases in active social media use in the study by Puukko et al.^42^, who followed 2891 adolescents over 6 years. However, no effect was found in the reverse direction. Additionally, Raudsepp^43^ collected EMAs data in 249 adolescent girls on three occasions, each 3 years apart, and found that initial depressive symptoms predicted sedentary behaviours, including Internet use, whereas the opposite was not true.

To summarize, existing literature reported small effects in both directions. Adverse effects are not surprising considering that adolescents who spend a lot of time online are likely to invest less time in activities beneficial for their well-being, like doing sports, doing homework, and sleep^44^. Additionally, they are exposed to content eliciting upward social comparison and envy, negatively affecting adolescent well-being^45,46^. However, if envy is perceived as “benign”, these mechanisms may also promote inspiration and motivational processes leading to positive well-being outcomes, as reported in a study with adults^47^. Positive effects may also derive from the enhancement of social connections and social capital^48,49^ through the use of social media, which is the most popular online leisure activity among adolescents^20^ and primarily done on one’s personal smartphone. Lower well-being can also increase the use of social media platforms to compensate for and escape from real-life problems^50^. Adolescents may find social support, release emotions, and search for online communities with similar interests to enhance their mood^51^. In addition, it has been suggested that digital technology use turn adolescents away from problematic behaviours such as violence outdoors^52^ and drug use, including alcohol consumption, since adolescents are already “constantly stimulated and entertained” by their smartphones^53^.

### The person-specific approach

Due to high levels of heterogeneity in the results of panel studies and meta-analyses, researchers have begun to explore person-specific effects^14^. It is likely that contradicting average effect sizes reported in large studies are due to the presence of substantial diversity in adolescents’ susceptibility to the effects of digital media use on well-being^54^. Indeed, within-person effects reported in longitudinal studies are stronger than individual effects, which may conceivably vary from highly positive to highly negative. Hence, to provide insight into individual unique susceptibility and to study how the effects vary from adolescent to adolescent, Beyens et al.^45^ introduced the person-specific approach, where the direction and magnitude of individual effects are estimated by collecting intensive longitudinal data using EMAs.

The few existing EMAs studies using this approach on adolescents focused on the association between social media and affective well-being^14^, also considering the active versus passive and private versus public social media use dichotomy^55^. For example, a recent analysis of 2155 real-time assessments^14^ collected during 1 week showed that the correlation between affective well-being and (passive) social media use in adolescents, including Instagram and WhatsApp usage, was very heterogeneous (with 44% of participants feeling neither better nor worse, 46% feeling better, and 10% worse). A follow-up study^55^ used EMAs for 3 weeks to assess if active and passive use of social media influenced well-being differently. However, sending messages did not make adolescents feel better than receiving instant messages or scrolling through others’ profiles. Instead, an adolescent reported positive, negative or no effects consistently across different activities and recipients, with the majority (45%) of participants not experiencing any positive or negative effect. The authors suggested that the presence of a null effect in a large portion of participants may be due to the possibility that positive and negative effects cancel out each other, thus resulting in a null effect.

To summarize, longitudinal results obtained so far suggested a mix of complex phenomena. The effects of digital media on young people’s well-being are *small*^15,22,31,39,42,56^, thus explaining little variation in well-being^57^, and vary from adolescent to adolescent^14^.

### Reciprocal and individual effects using trace data

Although previous studies (using both EMAs and traditional surveys) investigated within-person effects, they relied on self-reported information of smartphone or social media use. However, it is commonly acknowledged that self-reports are subjected to systematic biases, which include, among others, recall, estimation, and social desirability bias^58–60^. Recall bias, for example, is the result of cognitive burden and occurs when respondents use heuristic shortcuts to recall the duration and frequency of everyday behaviors^61^. Furthermore, problems with time estimation are common, especially among younger populations who are still developing a sense of time and the ability to quantify the time they engage in different activities^62^. In-situ assessments (i.e., EMAs) may help overcome recall biases by providing more accurate estimations of the time spent online with respect to retrospective survey methods^63^. However, they still show some limitations. In particular, when compared with objective trace data, time estimates of smartphone and social media apps usage collected through EMAs have been described as overestimated^64^. Hence, researchers started to investigate the frequency and duration of smartphone use by incorporating objective trace data^65^, though studies collecting such data in underage populations are still rare^64,66^. In line with the latter trend, the present study relied on trace data to estimate the duration and frequency of smartphone use, and analyzed it in relation to in-situ assessments of well-being. To note, combining behavioural and self-reported measures is beneficial to remove potential spurious correlations between self-reports: Self-reporting on two things may lead to correlation regardless of whether the two concepts are really correlated.

**Figure 1:**
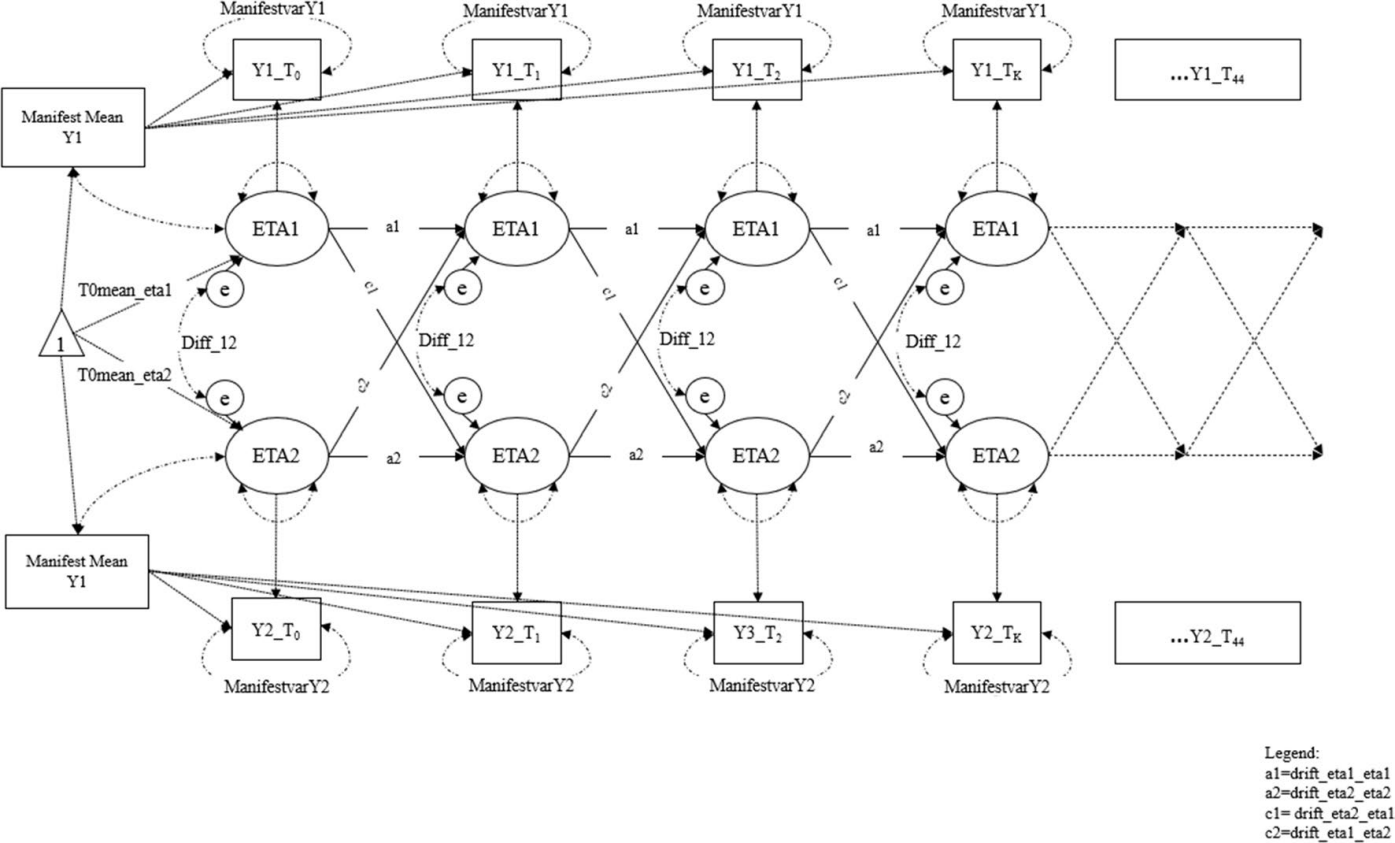
Graphical representation of a hierarchical Bayesian continuous time structural equation model with two manifest indicators (Y1 and Y2) measuring within-effects of two latent processes (ETA1 and ETA2). Legend: Y1 = Well-being; Y2 = Frequency/Duration ofsmartphone use; Manifest Mean = Continuous manifest intercept (Between-person component); Manifestvar = variance and covariance of manifest indicators (i.e., measurement error); eta1 = latent process of well-being; eta2 = latent process of frequency/duration of use; a1 = drift_eta1_eta1 (auto-effect of well-being); a2 = drift_eta2_eta2 (auto-effect of frequency/duration of use) c1 = drift_eta2_eta1 (cross-effect of well-being on frequency/duration of use); c2 = drift_eta1_eta2 (cross-effect of frequency/duration of use on well-being); Diff = covariance of the latent process. In the model, process intercepts are set to 1.00. Regressions and variances in the latent portion are all (after the first time point) conditional on the time interval.

Also, the investigation of bidirectional effects using intensive longitudinal data is still at the beginning. Classic, regression-based dynamic models—e.g. autoregressive and cross-lagged panel models, latent change scores, latent growth curve models—are generally used to investigate how psychological constructs fluctuate over time within a subject. However, they rely on the assumption that the intervals between measurements are the same^67^ and allow only limited random effects (e.g., for intercepts and slopes) without providing a full range of random parameters (e.g. temporal regressions and covariances) for single individuals. To overcome these limits, the present study used Hierarchical Bayesian Continuous Time Dynamic Modelling^68^ to model smartphone use and well-being as continuously changing and interacting processes, with system characteristics that vary across individuals^67,68^. To understand better these features, we can consider two extremes of a continuum in dynamic models. At one end, traditional panel models compute a single set of parameters describing the dynamics of all the individuals (“all leaves weigh exactly the same”). In other words, the estimated model assumes that the underlying dynamics in the psychological processes of all the subjects are the same. At the other end, person-specific time series (N = 1) analyses treat each participant as independent to the others (“one leaf is to another, as to a rock”^68^(p. 3)). However, large amounts of data are generally necessary for the estimation of person-specific time series parameters. The Hierarchical Bayesian Continuous Time Dynamic Modelling approach is in the middle of this continuum, allowing every individual to have their own parameters but also assuming some similarity across subjects according to estimated population distributions. Additionally, in *continuous time dynamic* models, autoregressive and cross-effects are modelled without any need to assume that time intervals between observations are equal or that processes only interact when observed. A graphical representation of the Hierarchical Bayesian Continuous Time Dynamic Model used in the present study is displayed in Fig. 1.

### The present study

This study aimed to investigate how well-being and smartphone use (i.e., frequency and duration) influence each other over time in a sample of adolescent participants. The study is part of the longitudinal MEDIATICINO panel study (www.mediaticino.usi.ch), investigating digital media use and youth well-being. In 2018, the panel study included 1419 students, of which 1374 (96.8%) completed a paper-and-pencil questionnaire at school. For 264 (18.6%) students, parents provided informed consent to invite their children to the intensive longitudinal study, during which data were collected via a dedicated application (called “Ethica”; ethicadata.com) installed on adolescents’ own devices. Despite parental consent, 169 (64%) students did not download Ethica. The remaining 95 students (6.9% of the initial sample) eventually participated in the Ethica study in 2018. All the data were collected through participants’ own devices for 45 consecutive days, for a total of 2548 assessments from day 1 to 45. The final analytical sample comprises 82 participants with complete and matched data (M_age_ = 13.47, ranging from 13 to 15 years, SD_age_ = 1.62, 54% females). Well-being was measured by asking participants how they felt on that day, with answers ranging from 0 “definitely not good” to 100 “definitely good”.

## Results

Participants provided valid trace data for an average of 35 days out of 45 (ranging from 4 to 45). ICCs showed that around 32%, 36%, and 39% of the variability in well-being, frequency, and duration of smartphone was due to between-person variance, respectively. The remaining percentage of variance can be explained by within-person fluctuations over time (i.e., intra-individual variability). Repeated-measure (within-person) correlations showed that frequency and duration of smartphone use were highly correlated (r = 0.451, p < 0.001), but no significant correlation was found between daily well-being and frequency (r = − 0.003, p = 0.887) or duration (r = 0.031, p = 0.178) of smartphone use.

### Well-being and frequency of smartphone use

Table 1 reports the results of continuous (i.e., time-independent) and discrete (i.e., time-dependent) time parameter estimates. Continuous drift parameters describe how the process *is changing*, whereas the discrete time parameters describe how the process looks *after* it has changed for some specific time period. To note, the interpretation of continuous auto-regressive effects should not be confused with the interpretation of discrete time parameters. In particular, the larger the positive effect, the stronger the downward pressure to come back to baseline after an increment. On the contrary, the larger the negative effect, the higher the upward pressure to come back to baseline levels after a depletion. Hence, positive continuous time auto-regressive parameters may result in negative discrete time auto-regressive parameters, and vice versa. The interpretation of continuous cross-regressions is similar to traditional cross-lagged effects, for example, the betas of a Random-Intercept Cross-Lagged Panel Model.

**Table 1:** Continuous and discrete parameter estimates of well-being and frequency of smartphone use.

Path	Continuous drift parameters	Discrete drift parameters			
	Estimate (95% CI)	1 day	3 days	7 days	30 days
**Auto-regressions**					
Well-being → Well-being (drift_eta1_eta1)	− 0.206 [− 0.303 to − 0.137]	0.817 [0.739 to 0.857]	0.552 [0.409 to 0.678]	0.258 [0.125 to 0.407]	0.005 [0.000 to 0.021]
Frequency of SP use → Frequency of SP use (drift_eta2_eta2)	− 1.764 [− 2.101 to − 0.145]	0.175 [0.124 to 0.238]	0.008 [0.002 to 0.019]	0.001 [− 0.001 to 0.005]	0.000 [0.000 to 0.0002]
**Cross-regressions**					
Well-being → Frequency of SP use (drift_eta2_eta1)	− 0.116 [− 0.093 to 0.320]	0.048 [− 0.039 to 0.135]	0.041 [− 0.034 to 0.119]	0.020 [− 0.014 to 0.066]	0.001 [0.000 to 0.003]
Frequency of SP use → Well-being (drift_eta1_eta2)	0.098 [0.008 to 0.191]	0.041 [0.003 − 0.082]	0.034 [0.003 to 0.073]	0.016 [0.001 to 0.039]	0.000 [0.000 to − 0.002]

*SP* smartphone. All effects not including the value of zero in the 95% CI interval were significant at the 0.05 level. Continuous drift parameters include continuous (i.e., time-independent) autoregressive and cross-lagged estimates in the drift matrix, Discrete drift parameters report the discrete (i.e., time-dependent) standardized effects at intervals of 1 day, 3 days, 7 days, and 30 days.

Population-level auto effects showed that changes in well-being (drift_eta1_eta1 = − 0.206, 95% CI [− 0.303 to − 0.137]) persist for a long period, i.e., they are closer to zero. Changes in the frequency of smartphone use (drift_eta2_eta2 = − 1.764, 95% CI [− 2.101 to − 0.145]) are more negative, indicating less persistence than seen for well-being. These results can also be understood in terms of the discrete time effects implied by the continuous time parameters: Well-being tends to predict itself the next days, as shown by the large auto-regressive effects (β_1day_ = 0.817, 95% CI [0.739–0.857]; β_3day_ = 0.552 [0.409–0.678]; β_7days_ = 0.258 [0.125–0.407]). However, after 30 days, the predictive power of well-being on itself declines (β_30days_ = 0.005 [0.000–0.021]). Auto-regressive values for the frequency of smartphone use are positive and of small size only for the day after (β_1day_ = 0.175, 95% CI [0.124–0.238]). Discrete time parameters become close to zero after few days (β_3day_ = 0.008 [0.002–0.019]; β_7days_ = 0.001 [− 0.001–0.005]; β_30days_ = 0.000 [0.000–0.002]), thus indicating that whatever changes in smartphone use do occur, they are only predictive for a few days.

**Figure 2:**
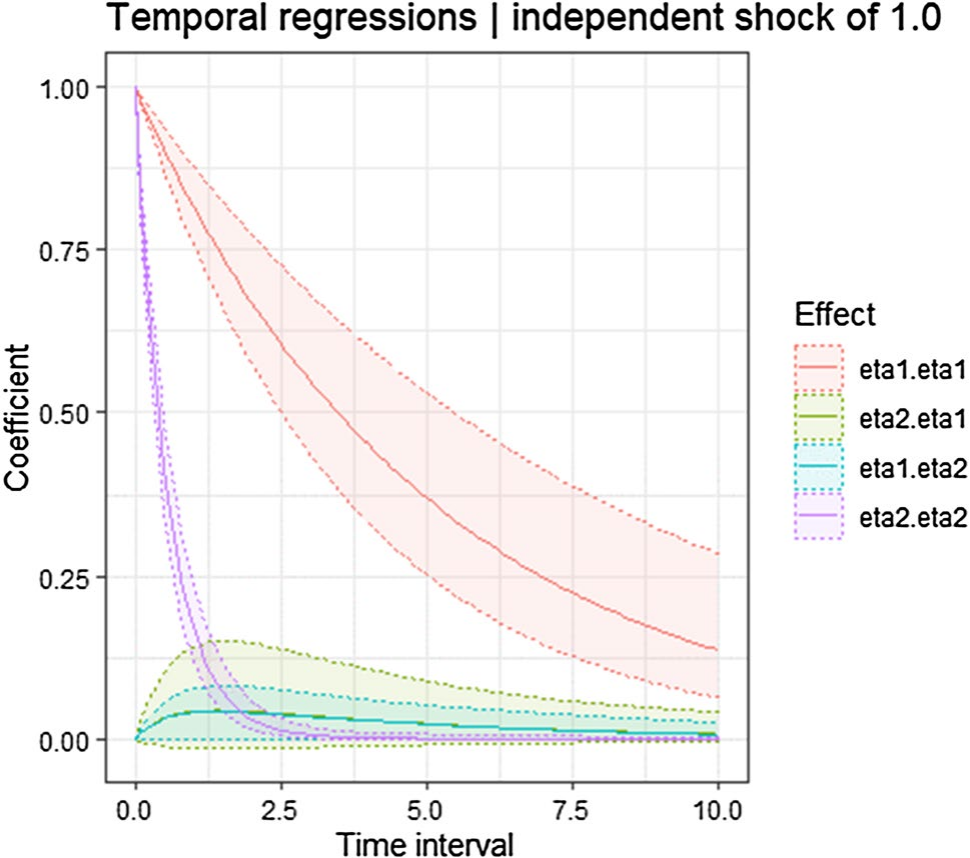
Representation of discrete time parameters of auto- and cross-effects of the Hierarchical Bayesian Continuous Time Dynamic Model of well-being and frequency of smartphone use. Legend: eta1.eta1 = auto-regressions of well-being; eta2.eta2 = auto-regressions of frequency of smartphone use; eta2.eta1 = cross-regressions of well-being on the frequency of smartphone use; eta1.eta2 = cross-regressions of the frequency of smartphone use on well-being.

**Figure 3:**
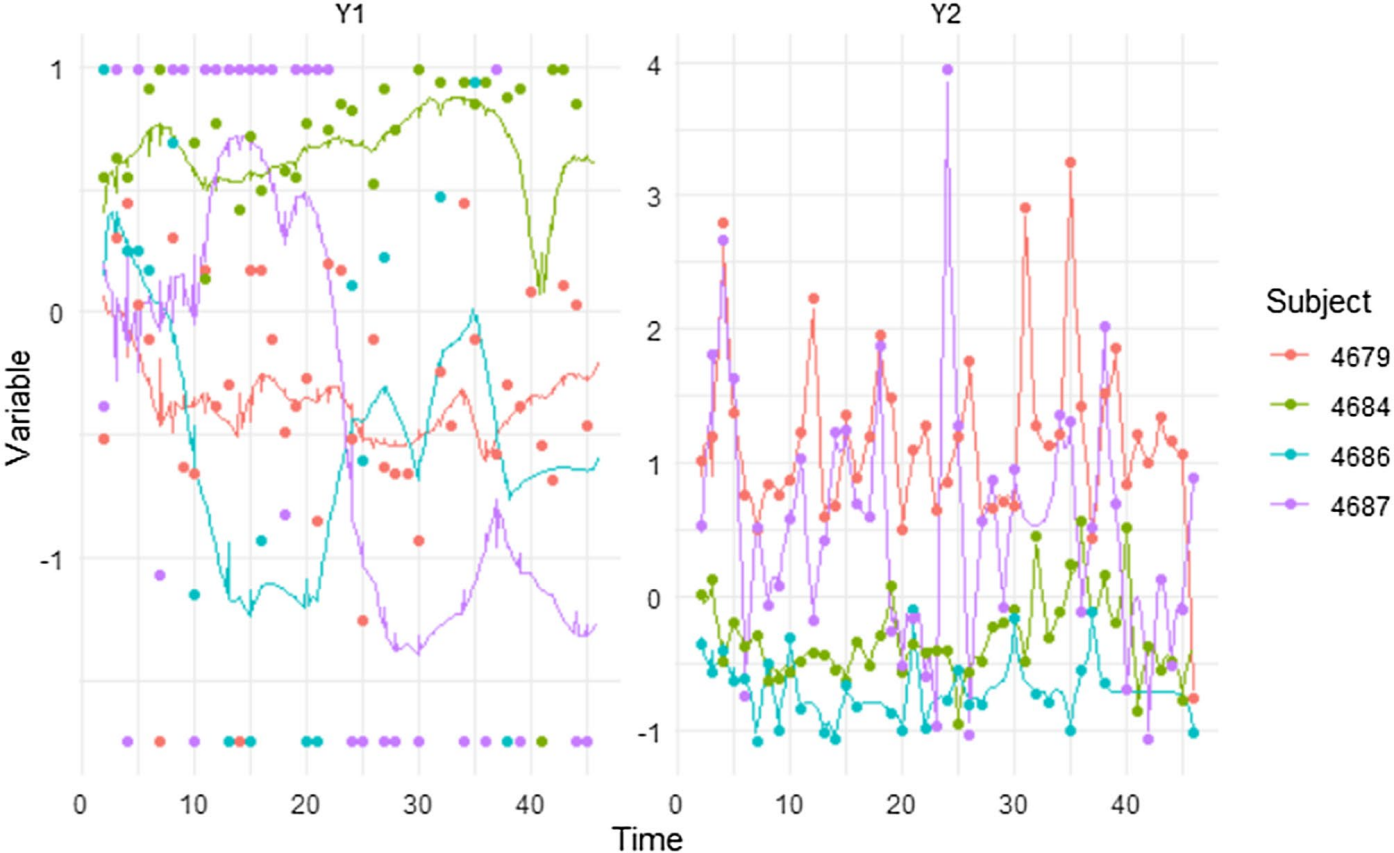
Observed data points of well-being (Y1) and frequency of use (Y2) for four specific subjects over 44 days. Auto-effects of Y1 are more stable over time, whereas Y2 showed more variation.

The continuous time cross-effect of the frequency of smartphone use on well-being is above zero. In other words, when smartphone use increases, well-being also likely follows (drift_eta1_eta2 = 0.098, 95% CI [0.008 to 0.191]). Cross-regressive estimates of well-being on the frequency of smartphone use are below zero, though not significant (drift_eta2_eta1 = − 0.116, 95% CI [− 0.093 to 0.320]). If they were significant, this result would suggest that changes in well-being negatively predict further changes in the frequency of smartphone use. These dynamics are also shown by the discrete cross-lagged effects: Higher frequency of smartphone use significantly predict higher levels of well-being the day after, although, according to the smallest effect size of interest (SESOI) in this study, the size of the effect is small (β_1day_ = 0.041, 95% CI [0.003–0.082]; β_3days_ = 0.034 [0.003–0.073]) and, after few days, non-existent (β_7days_ = 0.016 [0.001–0.039]; β_30days_ = 0.000 [0.000–0.002]). Higher levels of well-being do not predict a higher frequency of smartphone use for the same time frame. Figure 2 shows the discrete time auto- and cross-regressive parameters, and how they vary depending on time interval. Individual trajectories (representative of within-person heterogeneity) in well-being and frequency of smartphone use are displayed in Fig. 3, representing observed data for four different participants.

### Well-being and duration of smartphone use

In Table 2, continuous auto-regressive effects showed that changes in well-being (drift_eta1_eta1 = − 0.736, 95% CI [− 0.959 to − 0.549]) persist longer than changes in the duration of smartphone use (drift_eta2_eta2 = − 1.393, 95% CI [− 1.624 to − 0.189]). However, the parameters are negative, suggesting that they return to baseline levels at some stage. In particular, discrete time parameters showed that well-being tends to predict itself over time (β_1day_ = 0.487, 95% CI [0.389–0.584]; β_3days_ = 0.125 [0.064–0.207]; β_7days_ = 0.009 [0.002–0.026]; β_30days_ = 0.000). Duration of smartphone use showed positive auto-regressive effects (β_1day_ = 0.254, 95% CI [0.202–0.309]), which do not persist for a longer time (β_3days_ = 0.021, 95% CI [0.011–0.036]; β_7days_ = 0.001, [0.000–0.002]; β_30days_ = 0.000 [0.000–0.000]). Because of some variability in estimates of well-being dynamics depending on whether frequency or duration of smartphone use was part of the model, we also ran a combined model, with all three variables entered at once, as a sensitivity check. The size of auto-regressive effects of well-being were the following: β_1day_ = 0.629, 95% CI [0.624–0.635]; β_3day_ = 0.253 [0.246–0.260]; β_7days_ = 0.041 [0.039–0.044]; β_30days_ = 0.000 [0.000–0.000], and other substantive conclusions were unaltered (the output of these supplementary analyses is available at https://osf.io/kg76p/).

**Table 2:** Continuous and discrete parameter estimates of well-being and duration of smartphone use.

Path	Continuous drift parameters	Discrete drift parameters			
	Estimate [95% CI]	1 day	3 days	7 days	30 days
**Auto-regressions**					
Well-being → Well-being (drift_eta1_eta1)	− 0.736 [− 0.959 to − 0.549]	0.487 [0.389 to 0.584]	0.125 [0.064 to 0.207]	0.009 [0.002 to 0.026]	0.000 [0.000 to 0.000]
Duration of SP use → Duration of SP use (drift_eta2_eta2)	− 1.393 [− 1.624 to − 0.189]	0.254 [0.202 to 0.309]	0.021 [0.011 to 0.036]	0.001 [0.000 to 0.002]	0.000 [0.000 to 0.000]
**Cross-regressions**					
Well-being → Duration of SP use (drift_eta2_eta1)	0.250 [0.101 to 0.402]	0.088 [0.035 to 0.141]	0.039 [0.014 to 0.071]	0.003 [0.001 to 0.009]	0.000 [0.000 to 0.000]
Duration of SP use → Well-being (drift_eta1_eta2)	0.128 [0.025− 0.221]	0.045 [0.009 to 0.077]	0.019 [0.004 to 0.036]	0.002 [0.000 to 0.004]	0.000 [0.000 to 0.000]

*SP* smartphone. All effects not including the value of zero in the 95% CI interval were significant at the 0.05 level. Continuous drift parameters include continuous (i.e., time-independent) autoregressive and cross-lagged estimates in the drift matrix, Discrete drift parameters report the discrete (i.e., time-dependent) standardized effects at intervals of 1 day, 3 days, 7 days, and 30 days.

**Figure 4:**
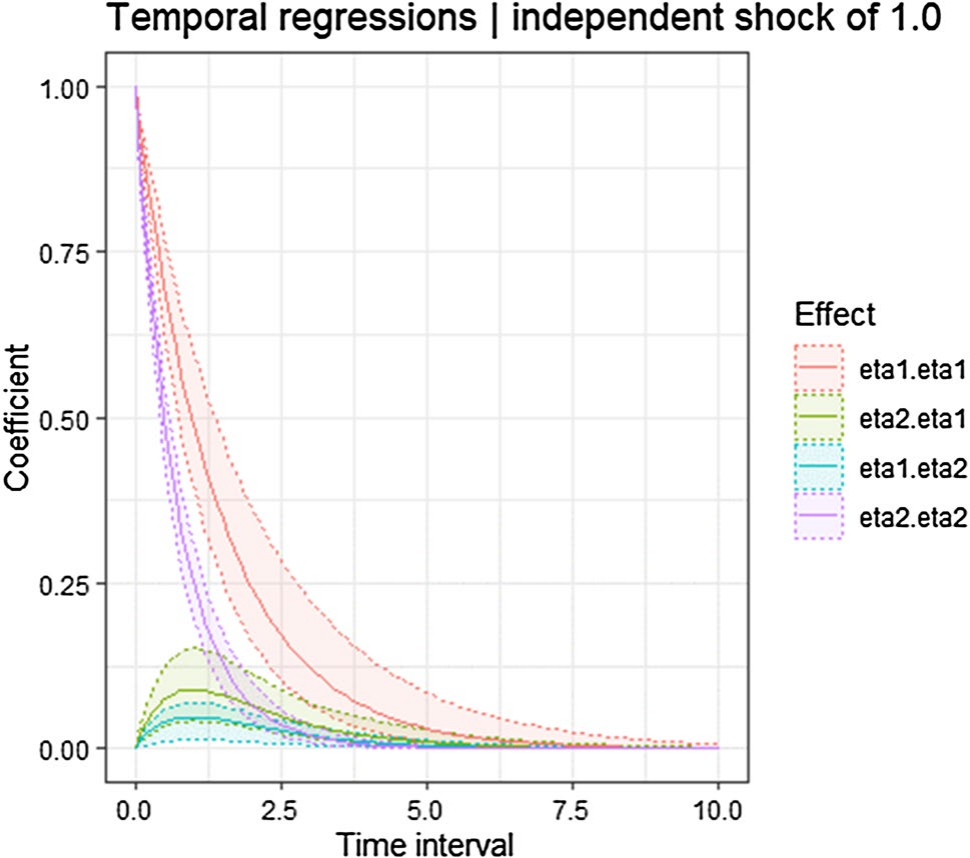
Representation of discrete time parameters of auto- and cross-effects of the Hierarchical Bayesian Continuous Time Dynamic Model of well-being and duration of smartphone use. Legend: eta1.eta1 = auto-regressions of well-being; eta2.eta2 = auto-regressions of duration ofsmartphone use; eta2.eta1 = cross-regressions of well-being on the duration of smartphone use; eta1.eta2 = cross-regressions of the duration of smartphone use on well-being.

**Figure 5:**
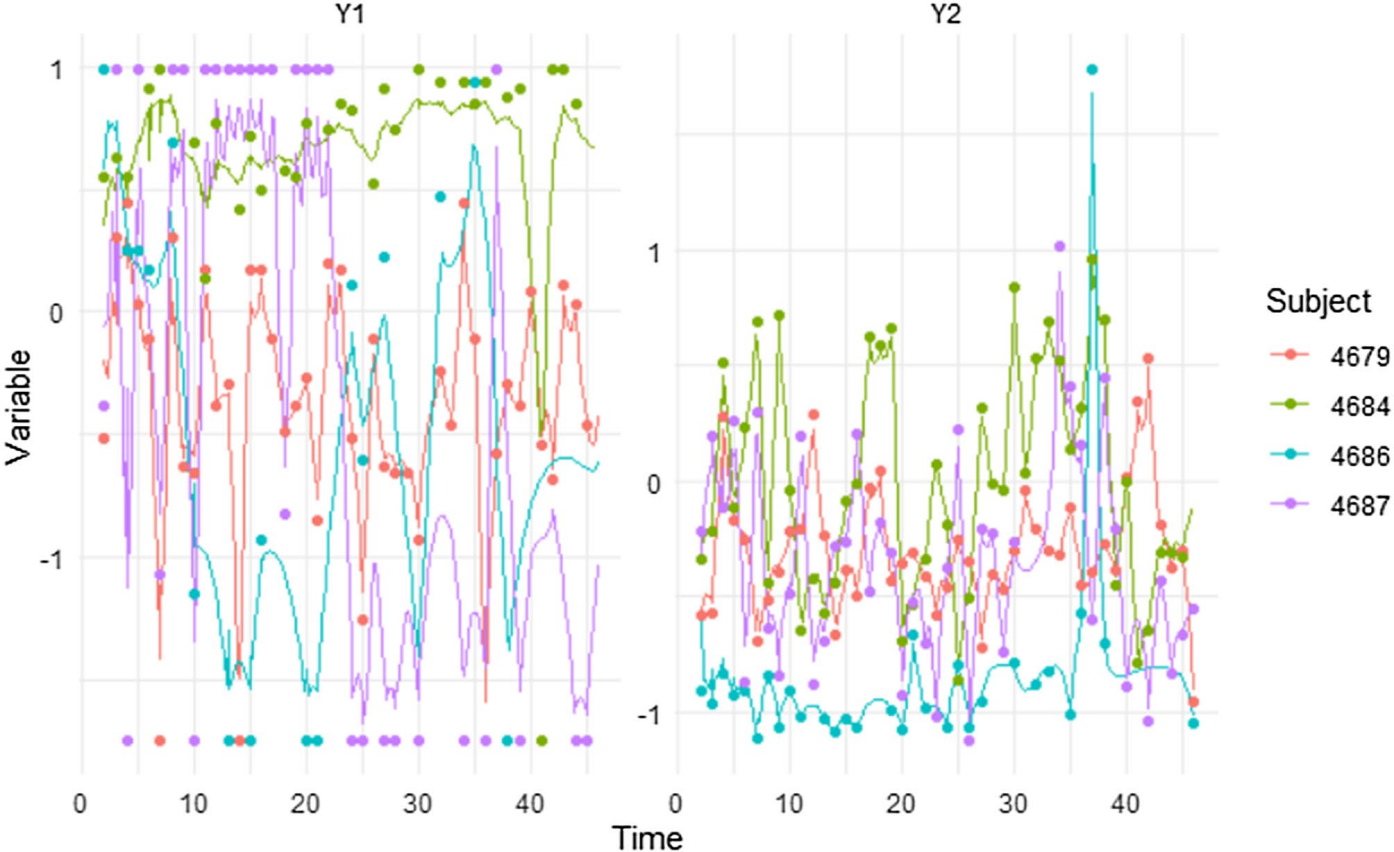
Observed data points of well-being (Y1) and frequency of use (Y2) for four specific subjects over 44 days. Auto-effects of Y1 are more stable over time, whereas Y2 showed more variation.

Continuous time parameters also showed that when the duration of smartphone use is high, well-being likely increases (drift_eta1_eta2 = 0.128, 95% CI [0.025 to 0.221]). Similarly, higher levels of well-being are followed by higher levels in the duration of smartphone use (drift_eta2_eta1 = 0.250, 95% CI [0.101 to 0.402]). In terms of discrete time cross-lagged effects, our results showed that higher levels of well-being predict a higher duration of smartphone use, though the effect is small (β_1day_ = 0.088, 95% CI [0.035–0.077]; β_3days_ = 0.039 [0.014–0.071]) with little persistence over time (β_7days_ = 0.003 95% CI [0.001–0.009]; β_30days_ = 0.000 [0.000–0.000]). Higher duration of smartphone use predicts higher levels of well-being during the next few days (β_1day_ = 0.045, 95% CI [0.009–0.077]), although the effect soon becomes non-existent according to our defined SESOI (β_3days_ = 0.019 95% CI [0.004–0.036]; β_7days_ = 0.002 [0.000–0.004]; β_30days_ = 0.000 [0.000–0.000]). Figure 4 represents the discrete time auto- and cross-regressive parameters and how they vary over time intervals. Both cross-lagged effects for these exemplary four subjects are estimated to be above zero. Individual trajectories (representative of within-person heterogeneity) in well-being and duration of smartphone use are displayed in Fig. 5, representing observed data for four different participants.

### Correlations between individual level parameters

When looking at correlations among individual level parameters of Model 1, adolescents with higher average levels in the frequency of smartphone use also showed lower cross-effects from the frequency of use to well-being (r_mm_Y2__drift_eta1_eta2_ = − 0.498, z = − 3.022). Additionally, in these adolescents, both changes in the frequency of smartphone use and well-being persist for long periods more stable over time, as represented by the positive correlations with the auto-effects (r_mm_Y2__drift_eta2_ = 0.362, z = 2.102; r_mm_Y2__drift_eta1_ = 0.348, z = 2.312). However, the frequency of smartphone use is more variable than well-being (r_mm_Y2__diff_eta2_ = 0.637, z = 5.317). On the other hand, adolescents with higher levels of well-being also reported less stable levels in this variable (r_mm_Y1__drift_eta1_ = − 0.425, z = − 3.212). Looking at Model 2, individuals with higher average levels in the duration of smartphone use showed more persistent changes in the same variable over time (r_mm_Y2__drift_eta2_ = 0.607, z = 5.289) and higher variability (r_mm_Y2__diff_eta2_ = 0.613, z = 4.472). On the contrary, adolescents with higher levels of well-being showed less persistent changes in well-being over time (r_mm_Y1__drift_eta1_ = − 0.285, z = − 2.435) and also lower cross-effects from the duration of smartphone use to well-being (r_mm_Y1__drift_eta1_eta2_ = − 0.431, z = − 2.018).

### Individual continuous time parameters

**Figure 6:**
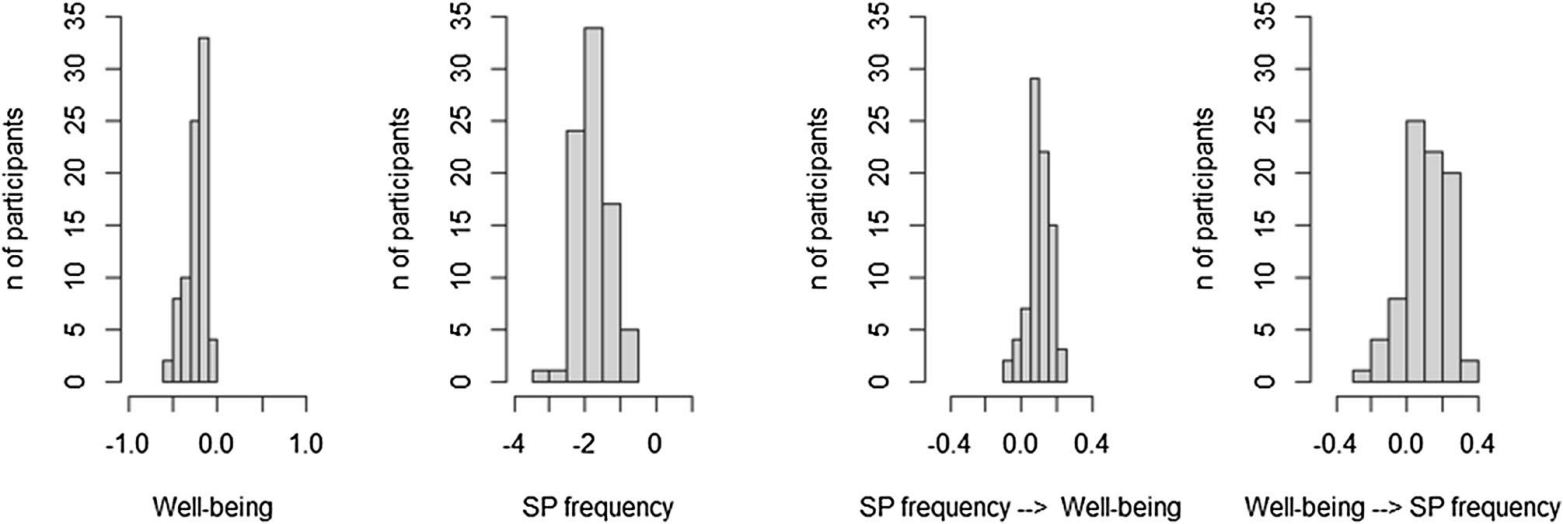
Distribution of individual continuous auto- and cross-effect parameters of the Hierarchical Bayesian Continuous Time Dynamic Model of well-being and frequency of smartphone use.

**Figure 7:**
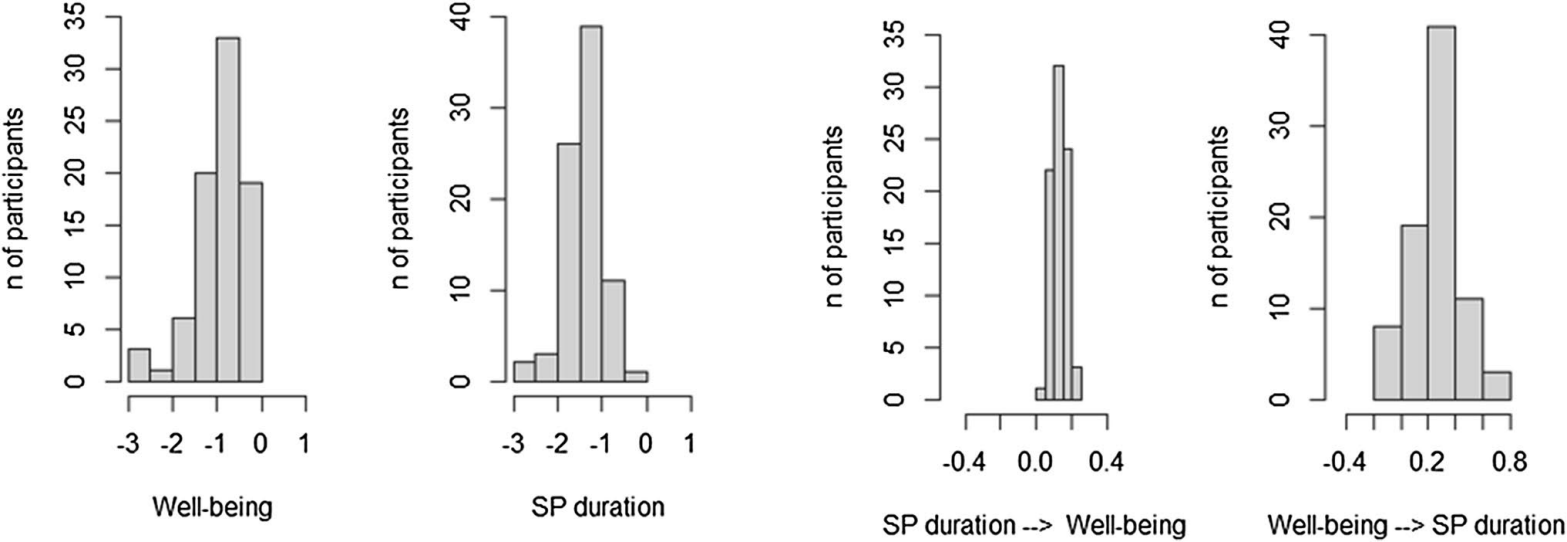
Distribution of individual continuous auto-and cross-effect parameters of the Hierarchical Bayesian Continuous Time Dynamic Model of well-being and duration of smartphone use.

Figures 6 and 7 present the distribution of individual continuous auto- and cross-effects of Model 1 and Model 2. Looking at the auto-effects of both models, all participants showed negative auto-effects for well-being, indicating that well-being tends to return to the baseline level after a change. In addition, all participants showed negative auto-regressive effects for the frequency and duration of smartphone use. Hence, after a period of less frequent or shorter smartphone use, the frequency and duration of smartphone use tend to return to baseline levels. The distribution of the auto-regressive effects parameters reflects the estimates for the total sample reported in Tables 1 and 2.

Individual continuous time cross-effects range from negative to positive. In particular, cross-effects of well-being on the frequency of smartphone use vary from − 0.291 to 0.356 (median = 0.105, IQR = 0.038–0.202). Similarly, when the frequency of smartphone use predicts well-being, cross-effects range from − 0.077 to 0.218 (median = 0.099, IQR = 0.065–0.145). Cross-regressive values from well-being to the duration of smartphone use vary from − 0.179 to 0.059 (median = − 0.079, IQR = − 0.107 to − 0.052). Whereas, they are all positive when looking at the opposite direction, i.e. duration of smartphone use influencing well-being, and they range from 0.045 to 0.227 (median = 0.125, IQR = 0.099–0.157).

## Discussion

The present exploratory study used daily assessments of well-being combined with trace data for the duration and frequency of smartphone use to disentangle the reciprocal and individual dynamics among the two variables in a sample of early adolescent participants. By doing so, this study simultaneously addressed limitations of previous research relying on self-report data and focusing on between-person correlations or one-sided longitudinal effects. We obtained four major findings to be discussed: (i) when participants used the smartphone more frequently and for a longer time, they also reported higher levels of well-being; (ii) well-being positively predicts subsequent duration of smartphone use; (iii) usage patterns and system dynamics showed heterogeneity, with many subjects showing reciprocal effects close to zero; and, finally, (iv) changes in well-being tend to persist longer than changes in the frequency and duration of smartphone use.

The first main finding indicates that the frequency and duration of user-device interactions might enhance participants’ well-being, although the effect might be small. Hence, easy access to the smartphone may give positive rewards to the user: Indeed, research showed that adolescents develop the unconscious habit to unlock their smartphones’ screen every 5 min^2^. This habit may be the result of deficits in self-observation (i.e., awareness) and self-reaction (i.e., control)^69^. Nowadays, social networking is listed as the most popular online leisure activity among Swiss adolescents^20^, meaning that the great majority of (habitual) smartphone use is dedicated to this activity. The cues provided by social networking applications through sophisticated algorithms, which determine the receipt of notifications and page layout, as well as individual (e.g., mood) and contextual (e.g., commuting time) factors, facilitate a habitual use of the smartphone. Repeatedly used applications like social media and instant messaging platforms^65^ foster the experience of short-term gratifications from incoming notifications, messages, and Likes, to the point that motivations for smartphone use are substituted by habitual use^70^. In fact, according to a recent scoping review on neuroscientific studies, online activities produce strong rewards for the brain, thus fostering subsequent use to seek short-term gratifications^71^.

A constant connection with the social sphere is often carried out by using lightweight messages, which enable a sense of co-presence among peers^72^ and allow communication with whom they interact less frequently in offline situations^48^. Indeed, positive effects may be related to the possibility to find more help on social media and connect with like-minded people^73^. A meta-review^21^ reported that, although the effect of digital media use on well-being was generally negative and small, when the type of usage involved active social interactions, it was positively related to well-being. Furthermore, a recent study^74^ analyzed data from 10,560 Facebook users and reported that authentic self-expression (versus self-idealizing) posting behaviours on social media were related to higher life satisfaction. Similarly, Gonzales and colleagues^75^ found that more meaningful text-based interactions through social media instant messaging applications were associated with higher self-esteem with respect to face-to-face or call-based communication; although, the effects were overall very small and lasted for few days. Along this line, a review suggested that digital media use is more likely to affect short-term positive and negative outcomes of well-being than long-term ones^73^. The ephemeral nature of these effects is in line with previous findings supporting “positive—yet fleeting—emotional experiences” resulting from social media posting activities, both in terms of mood-boosting and bridging social capital^76^. Considering the magnitude and the temporal dynamics of these relationships, it is possible that *ephemeral effects result in null or mixed effects* when investigated in traditional panel studies and summarized in meta-analyses. However, one should also bear in mind that our results represent changes on an approximately daily level. That said, changes in the usage patterns over, for example, years could result in a very different pattern of results. Indeed, a recent study pointed out that state-like measures of well-being may influence the same trait-like constructs^77^. Thus, one can argue that short-term effects may accumulate over time. However, we do not know yet how these ephemeral effects due to digital media use may influence long-term well-being outcomes.

Secondly, we found small positive effects from well-being to the duration of smartphone usage. This effect may mirror the (social) needs that smartphone use fills in many aspects of adolescents’ lives^78^, including the enhancement of emotions, search for enjoyment, or coping with stressful life events as explicated in the model of compensatory internet use^50^. It is possible that adolescents who feel better are also more inclined to share their whereabouts and self-disclose information through smartphone-mediated communication. In particular, a positive loop between duration and well-being may be at the base of the reinforcing loop hypothesized by Brand et al.^79^, which is also the starting point of online addictive behaviours.

Finally, our results mirror the ones of previous studies focusing on well-being^14,55^, together with similar studies exploring how social media use influences the experience of envy, inspiration, and enjoyment^80^, friendship closeness^81^, self-esteem^82^, and distraction^83^. In general, these studies indicated that each individual shows positive, negative, or, mostly, null effects. Similarly, we also found heterogeneity when looking at individual continuous time cross-effects. For example, in participants who used the smartphone more frequently, subsequent levels of well-being did not tend to change. Additionally, adolescents with higher levels of well-being were less influenced by longer duration of smartphone use. Regardless of the effect size, complex psychological phenomena are determined by many causes, so it is likely that any individual cause has only a small effect, which, however, should be acknowledged^84^. As recently stated by Götz et al.: “we must focus on the interplay of many tiny causes working alone and in concert, with each individual cause playing a smaller individual role than we previously may have thought. Thus a nuanced consideration […] of small effects can yield important theoretical advances that would otherwise be missed”^84^^(p.6)^. Additionally, not only can we say that: “No screen time is created equal; different uses will lead to different effects”^(^^73^^, p. 139)^, but we should also acknowledge that smartphone, and digital media use in general, do not show a linear dose–response relationship^41,73^. Hence, considering individual dynamics is becoming more crucial to understanding the daily and continuous experience with digital technologies. The present study shows that this temporal analysis can be done by modelling the relationship between media use and well-being beyond mere linear correlations happening in discrete time windows.

The last finding, i.e. changes in well-being tend to persist over time, mirrors results obtained in longitudinal studies investigating the stability of well-being in adolescence^85,86^. It is also in line with findings from EMAs studies investigating (affective) well-being on a daily basis^77,87^. On the contrary, duration and frequency of smartphone use showed a greater variation on a daily basis and across different days, with auto-effects being very small and reaching a null value after few days—especially in the case of the frequency of smartphone use. That said, changes in smartphone use offer no predictive usefulness after a few days. In line with data on smartphone use obtained by screenoms^88^ (screenshots of a smartphones’ screens taken every 5 s while used), we can sustain what the authors suggested: “individuals’ smartphone use is non-continuous, fragmented, and scattered throughout the day in irregular and idiosyncratic ways”^(p. 5)^. Hence, when modelling smartphone use, we should consider that the extent of within-person variability in temporal dynamics would make it difficult to interpret an average trajectory at the population level in a useful way since it would not represent any specific subject’s behaviour. That said, the estimation of individual parameters prevents the ecological fallacy^89^, which is committed when one assumes that effects found for the ‘hypothetical average’ individual are true for everyone, i.e. when an inference is made about a particular subject based on aggregate data^90^. As pointed out by the new research trend called *digital phenotyping*, it is now crucial to study the “moment-by-moment quantification of the individual-level human phenotype in situ using data from personal digital devices, in particular smartphones”^91^^(p3)^ to form a picture on each individual lived experience including behaviours and psychological states. At the same time, we also acknowledge that some authors warranted about the person-centred approach in studying media effects, by stating that “prioritizing variation over interpreting and understanding average associations risks atomizing associations”^92^^(p.4)^, and that would go in stark contrast with the main aim of *social* sciences. Here, we showed that it is possible to consider and estimate, at the same time, both the population- and the individual-level effects, by incorporating the strengths of each approach into a single, advance statistical analysis. By doing so, social scientists can interpret both the average and the person-specific results and rely on that to test future hypotheses more precisely and describe behavioural and psychological processes in a more sophisticated and (ecologically) valid way. Hierarchical approaches (as used herein) that allow for heterogeneous individual trends and parameters, while still offering an overall or population level estimate, offer a nice resolution—they help distinguish “heterogeneity of the effects” (reflecting between-person variation, i.e. individual parameters) from “uncertainty around the mean”^92^ (describing the variability of the average relationship from sample to sample, i.e. the confidence interval around the fixed effect).

### Limitations and future directions

We acknowledge that the present study has several limitations. First, we only looked at one general operationalization of well-being, but smartphone use can be related with other well-being facets, classified as part of the “psychopathology” or “psychological well-being” dimensions^21^, where the absence of one does not implicate the presence of the other. Hence, future studies should look at these facets over time and how they are related to smartphone use at both trait- and state-like levels.

Additionally, we did not include data on the type of usage considering different apps and contents. Although gathering such data is challenging, they are now pivotal to better comprehend which contents may elicit bigger/smaller effects on well-being and vice versa. Furthermore, we mainly relied on a “technology-centred” approach by describing the frequency and duration of smartphone use; however, a “user-centred” approach would complement the data by asking participants about their subjective experiences like attitudes, motivations as well as negative or positive perceptions of the content consumed^21^.

Concerning the study sample, only a small percentage of participants already enrolled in the MEDIATICINO panel study joined the app-based Ethica study. Due to restrictions from the collaborating schools, we could not recruit individuals directly, helping them to install the Ethica app and register to the study. This was all done via an information leaflet and a video tutorial. However, enrollment of participants and their adherence are huge challenges in EMAs studies involving adolescents. We suggest that using direct contact with the study participants, guidance in downloading the tracking application, good incentives, and giving feedback would allow future researchers to avoid such issues^93^. The present study results should not only be replicated with larger samples but also with samples including more vulnerable populations, e.g. with psychopathological problems or coming from low socio-economic backgrounds. Also, adolescents’ age may moderate the effects since older adolescents have reported less negative outcomes^21^, hence different age ranges should also be considered.

## Conclusions

To conclude, the small but positive relations seen in our results suggest that smartphone use, broadly speaking, is unlikely to lead to reductions in well-being, at least in the short term—indeed, participants reported higher well-being after having used smartphones more frequently and for a longer time. However, the effects were “ephemeral” and of small size. The ephemeral nature of these effects may additionally explain the contradictory literature on media effects, which mainly described small and inconsistent findings. In this regard, short-term and highly rewarding effects may accumulate through time and at scale, which would lead to a cumulative media effects science built on small effects^84^. The long-lasting effects of small, ephemeral effects should be further explored in future studies using an intensive longitudinal design coupled with traditional longitudinal panel studies. To note, smartphone use is highly idiosyncratic, hence, the day-to-day assessment is essential in order to capture the unique experience of each adolescent using the smartphone.

## Methods

### Procedure

The present study is part of the longitudinal MEDIATICINO panel study (www.mediaticino.usi.ch), which relies on an annual self-administered paper-and-pencil questionnaire distributed among approximately 1400 adolescents living in Canton Ticino, Italian-speaking Switzerland. The survey data are collected in collaboration with schools. Anonymity is maintained by using a Unique Identifier (U-ID) associated with the student name, to which only school staff has access when distributing the questionnaires. More details on the data collection procedure can be found elsewhere^94^. Only participants with parental consent received further information on how to download the application and register for the study. With respect to students who did not join the Ethica study, participants did not report any difference in gender (p = 0.205), perceived socio-economic status (p = 0.229), or self-reported daily smartphone use (p = 0.114). However, differences were found in parents’ surveys, with parents who reported higher levels of smartphone use (p < 0.001) and smartphone addiction (p = 0.009) being more likely to give their consent for the Ethica study^95^. Ethica is available for both Android and iOS operating systems. Once registered, the app automatically gathers data on smartphone use, such as screen time and battery status. Ethica also allows sending in-situ surveys to participants. A generated login e-mail address matched to participants’ U-ID was used to combine digital trace data with self-report data.

### Ethical considerations

Participant recruitment through schools and data collection were carried out following the guidelines and regulations of the Cantonal education administration of Ticino, who approved the annual panel study based on self-report questionnaires and the embedded Ethica study. The Ethica study also received approval from the Ethics Committee of the Università della Svizzera italiana, where the research was carried out, and from the Cantonal Data Protection Officer of Ticino. Participation in the Ethica study required active consent by parents. Students provided their active consent directly in the Ethica application upon enrolment. To guarantee students’ anonymity, a generated login e-mail address required to enrol in the Ethica study was matched to participants’ U-ID.

### Measures

*Well-being* was measured by asking participants the following question once a day: “How do you feel today?”. Surveys were randomly sent in the evening, between h18 and h20.30 (to avoid interference with school hours). Response options ranged from 0 “definitely not good” to 100 “definitely good” on a visual analogue scale (M = 64, SD = 36.57). When combining digital traced data and self-report data, missings in self-report measures of well-being were around 24%. A full information approach within ctsem package was used to filter missing data.

*Duration and frequency of smartphone use* were automatically traced via Ethica on participants’ devices for 45 consecutive days from the enrollment date. Hidden Markov Models were used to handle missing data^96^. Participants spent, on average, 157 min per day on their smartphones (median = 124 min, IQR = 55–210 min). The frequency of smartphone use was assessed by counting how many times participants turned on their smartphone screens during a day. In general, participants turned on their devices for 63 times per day (median = 48, IQR = 23–87).

### Data analysis

Before conducting the main analysis, outliers and cases with no variability were removed. After the initial check, four cases were removed due to extreme values in the traced data (e.g., traced duration > 15 h/day). Another seven cases were removed due to a lack of variability in well-being and traced data (SD = 0 or not computable because the number of observations was ≤ 2). Two participants downloaded the application but did not provide any data. Intraclass Correlation Coefficients (ICCs) were estimated for each outcome variable to partition the variation due to individual differences. Repeated-measure correlations were calculated to estimate if well-being, traced frequency, and duration of smartphone use were related to each other in the same subject.

Next, a Hierarchical Bayesian Continuous Time Dynamic structural equation model was implied using the R package “ctsem”^67,97^. To estimate how the process is changed after a particular timeframe, discrete time parameters were computed for different time lags. Hierarchical Bayesian Continuous Dynamic Models allow individual variation in all parameters across subjects (random effects). The latent dynamic model (with temporal effects contained in the drift matrix) includes auto- and cross-effects, where negative values on auto-regressive effects should be interpreted as the tendency of the process to return to the baseline (dissipation) as time goes by, whereas positive values indicate an explosive process where deviations from baseline accelerate rather than dissipate. Similarly, positive cross-effects indicate that the other tends to follow as one process goes in a positive direction. In contrast, negative cross-effects indicate that when one process is above baseline, the other tends to decrease.

In interpreting within-person, discrete time effects, we set the smallest effect size of interest (SESOI)^98^ at β = 0.05 as reported by the study of Beyens et al.^99^, and as suggested by a recent meta-review^21^. Hence, we considered all effects ranging from − 0.05 to 0.05 as non-existent to very small, whereas β larger than 0.05 and − 0.05 were interpreted as positive and negative effects, respectively.

### Model building procedure

To aid the transformation and computation of priors, data were scaled and grand mean centred before the main analysis. The first step is the model specification. We specified two latent variables (frequency resp. duration of smartphone use and well-being) which were measured by two manifest indicators (one for each latent variable). A 2 × 2 diagonal LAMBDA matrix was specified and included each latent variable auto-effect on itself (called “drift_eta1_eta1” and “drift_eta2_eta2”, positioned in the diagonal of the matrix), and each cross-effect of one variable at Time_k−1_ on subsequent levels of the other variable at Time_k_ (called “drift_eta1_eta2” and “drift_eta2_eta1”, the off-diagonals). The direction of the effect should be interpreted from column to row (e.g., where drift_eta1(row)_eta2(column) is read as the effect of change in eta2 on later values of eta1).

In our case, a continuous time dynamic model was specified, leaving parameters of latent processes and the measurement model free, except for process intercepts (set to 1.00). The model was fitted with a maximum a posteriori approach and optimization with priors (in addition, a narrow prior for the estimation of SD of non-intercept random effects was included to allow for individual variability across all parameters without making model estimation overly difficult). Since we were interested in dynamics over different time frames, we computed discrete time parameters for 1 day, 3 days, 1 week (7 days), and 1 month (30 days). Next, individual parameters were extracted. The model matrix is reported in Fig. 3 of the “Appendix”. For more details on model specifications, see Driver and Voelkle^68^.

Once the parameters had been estimated for each subject, we reported descriptive results for auto- and cross-effects. The R code used for the analyses, including the output of the main and combined models, is freely available at https://osf.io/kg76p/. The results of the Hierarchical Bayesian Continuous Time Dynamic Model are reported as follows. Parameters for each model include means and posterior intervals of the population distribution, i.e., the uncertainty around the fixed effects (see Table 1 in the “Appendix”), standard deviations and posterior intervals of individual parameters with respect to the population mean values, i.e., the heterogeneity of the effects (see Tables 2 and 4 in the “Appendix”), correlations and posterior intervals of the random effects (see Tables 3 and 5 and Figs. 1 and 2 in the “Appendix”), and model matrix (see Fig. 3).

## Supplementary Information

Supplementary Information.
