# Supplementary material for: Dynamics of adolescents’ smartphone use and well-being are positive but ephemeral
Source: Sci Rep. 2022 Jan 25;12:1316. doi: 10.1038/s41598-022-05291-y (PMC8789843; doi:10.1038/s41598-022-05291-y)
Supplement: Supplementary file 1 — Supplementary Information. [file 41598_2022_5291_MOESM1_ESM.docx]

**Appendix**

**Well-being and frequency of smartphone use.** The means of the population distribution represent the estimated mean of the parameter distribution and the uncertainty around the fixed effects (see Table 1). T0means are the initial stage of the latent processes with respect to later stages. Hence, positive values indicate that initial levels of well-being and frequency of smartphone use are higher than at later stages, i.e. they diminish as time goes by. This was especially evident for well-being measures (T0m_wellbeing= .287, 95%CI [.053 to .524]) but less for frequency of smartphone use (T0m_frequency=.078, 95%CI [-.174 to .319]).

In Table 1, the asymDIFFUSION covariance matrix represents the latent process variance and covariance. It shows that well-being has less variation over time (asympDIFF_eta1=.345), with respect to the frequency of smartphone use (asympDIFF_eta2=.421), which is in line with the inconsistency found in the auto-effect. Covariance parameters do not show any relationship (asympDIFF_eta2_eta1=.001). Variance in the manifest indicator (mvar) reflects within-person variability in measurement error, and it is higher for well-being, indicating that measures of well-being tend to fluctuate more due to, for example, measurement limitations and short-term situational factors. This is in line with the self-reported nature of the measure, whereas objectively traced data of smartphone showed less measurement variation. Manifest means (mm) show the continuous intercepts of manifest indicators, reflecting between-person differences in baseline levels.

Standard deviations of individual parameters (Table 2) show how individual values differ from the population mean values – i.e. between-subject variance – thus reflecting the heterogeneity of the effects. Individual differences in T0means represent diversities in the initial level of the latent processes and show that frequency of smartphone use has more variation in the initial latent process with respect to levels of well-being (SD_T0m_eta1_ = .509 versus SD_T0m_eta2_ = 1.140). Variation in auto-effects mirrors how individuals vary in the persistence of change. In this case, the auto-effect of the frequency of smartphone use show a larger variation over time with respect to well-being (SD_drift_eta1_=.479 versus SD_drift_eta1_=.549). Additionally, variation in cross-lagged effects shows that well-being influences the frequency of use with more variation (SD_drift_eta2_eta1_=.210) than the opposite (SD_drift_eta2_eta1_=.082). Between-subjects variation in diffusion and manifestvar parameters reflect individual differences in the variance/covariance of the latent process and measurement error, respectively. Variation in the latent process of the frequency of use is higher than well-being, however, assessment of the latter is prone to higher measurement error. Finally, manifest means showed between-subject variation in the manifest intercepts, with higher differences in well-being levels (SD_mm_Y1_= .672 versus SD_mm_Y2_=.545). Table 3 represents correlations among individual subject parameters.

**Well-being and duration of smartphone use.** T0means indicate that levels of well-being were higher at the initial stage than at later stages. In other words, they diminish as time goes by (T0m_wellbeing= .350 95%CI [.161 to .523]; whereas the duration of smartphone use augmented (T0m_duration=-.171, 95%CI [-.306 to -.029]). The asymDIFFUSION covariance matrix in Table shows that well-being has a similar variation (asympDIFF_eta1=.466) of the duration of smartphone use (asympDIFF_eta2=.411). Covariance parameters show a small positive trend, reflecting that as one variable augments (i.e., well-being), also the other does (i.e., duration of smartphone use) (asympDIFF_eta2_eta1=.021). Variance in the manifest indicator (mvar) is higher for well-being (mvarY1=.336), indicating that measures of well-being tend to fluctuate more (i.e., showing more measurement error) than the duration of use (mvarY2=.062). Manifest mean (mm) of the continuous intercept for well-being is lower than the duration of use.

Standard deviations (Table 4) of individual subject parameters in T0means show that well-being levels display more variation in the initial latent process (SD_T0m_eta1_=.759 versus SD_T0m_eta2_=.640). Variation in auto-effect mirrors how individuals vary in the persistence of change. The auto-effect of well-being (SD_drift_eta1_=.884 versus SD_drift_eta2_=.449) also shows a larger variation over time with respect to the duration of smartphone use. Additionally, variability in the cross-effect from well-being to the duration of use is higher than the opposite (SD_drift_eta2_eta1_=.264 versus SD_drift_eta1_eta2_=.053). Variation in the latent process of well-being is higher than the duration of use, as well as its assessment is more prone to measurement error. Finally, manifest means show higher between-subject variation in the manifest of well-being. Table 5 represents correlations among individual subject parameters.

**Table 1.** Population means and posterior intervals of parameters of Hierarchical Bayesian Continuous Time Dynamic Models (=uncertainty around the fixed effects).

|  | **Population mean values**  **(Model 1– Well-being and smartphone fequency)** | | | | | | **Population mean values**  **(Model 2– Well-being and smartphone duration)** | | | | |
| --- | --- | --- | --- | --- | --- | --- | --- | --- | --- | --- | --- |
| **Parameter** | **mean** | **sd** | **2.50%** | **50%** | **97.50%** | **mean** | | **sd** | **2.50%** | **50%** | **97.50%** |
| T0m_eta1 | 0.2865 | 0.1195 | 0.0534 | 0.2813 | 0.5243 | 0.3502 | | 0.0925 | 0.1607 | 0.3511 | 0.5235 |
| T0m_eta2 | 0.0783 | 0.1268 | -0.1741 | 0.0825 | 0.3192 | -0.1712 | | 0.0717 | -0.3063 | -0.1711 | -0.0292 |
| asympDIFF_eta1 | 0.345 | 0.0506 | 0.2595 | 0.3404 | 0.4576 | 0.4666 | | 0.0563 | 0.3642 | 0.4613 | 0.5902 |
| asympDIFF_eta2_eta1 | 0.0014 | 0.0167 | -0.03 | 0.0019 | 0.0355 | 0.0208 | | 0.0167 | -0.0102 | 0.0208 | 0.0543 |
| asympDIFF_eta2 | 0.4215 | 0.037 | 0.3511 | 0.4193 | 0.5007 | 0.4109 | | 0.0344 | 0.3509 | 0.4096 | 0.4812 |
| mvarY1 | 0.6408 | 0.0378 | 0.5688 | 0.6395 | 0.7191 | 0.3358 | | 0.0571 | 0.2402 | 0.3329 | 0.4652 |
| mvarY2 | 0.0665 | 0.0443 | 0.0159 | 0.0563 | 0.1762 | 0.0626 | | 0.0396 | 0.0158 | 0.0545 | 0.165 |
| mm_Y1 | -0.201 | 0.1045 | -0.4039 | -0.2032 | -0.0053 | -0.2604 | | 0.0777 | -0.405 | -0.2644 | -0.0977 |
| mm_Y2 | -0.0382 | 0.0608 | -0.1571 | -0.0374 | 0.0841 | -0.15 | | 0.0566 | -0.2568 | -0.1513 | -0.0425 |

Legend: eta1=latent process of well-being; eta2=latent process of frequency/duration of use; T0m_ = latent process mean at first time point; asympDIFF= variance/covariance of the latent process; Y1= Well-being; Y2=Frequency/Duration of smartphone use; ); mvar=variance and covariance of manifest indicators (i.e., measurement error); mm_= Continuous manifest intercept

**Table 2**. Population standard deviations and posterior intervals of the Hierarchical Bayesian Continuous Time Dynamic Models model of well-being and frequency of smartphone use. This table shows how much individual parameters tend to differ from population mean, i.e. the unexplained between-person variance (=heterogeneity of the effects).

| **Variable** | **Mean** | **Standard deviation** | **Confidence intervals** | | |
| --- | --- | --- | --- | --- | --- |
|  |  |  | **2.50%** | **50%** | **97.50%** |
| T0m_eta1 | 0.5096 | 0.0894 | 0.3628 | 0.4977 | 0.7087 |
| T0m_eta2 | 1.1402 | 0.095 | 0.958 | 1.1387 | 1.3425 |
| drift_eta1 | 0.4798 | 0.0558 | 0.3704 | 0.4788 | 0.5976 |
| drift_eta1_eta2 | 0.082 | 0.0251 | 0.0396 | 0.0809 | 0.1381 |
| drift_eta2_eta1 | 0.2102 | 0.055 | 0.1015 | 0.2082 | 0.3159 |
| drift_eta2 | 0.5493 | 0.1296 | 0.305 | 0.5438 | 0.8208 |
| diff_eta1 | 2.1939 | 0.2996 | 1.7215 | 2.1566 | 2.843 |
| diff_eta2_eta1 | 0.0216 | 0.0289 | 0.0005 | 0.0086 | 0.1071 |
| diff_eta2 | 2.2721 | 0.3833 | 1.6436 | 2.2404 | 3.1006 |
| mvarY1 | 1.1317 | 0.2575 | 0.7702 | 1.0829 | 1.7633 |
| mvarY2 | 0.1601 | 0.2024 | 0.0054 | 0.0794 | 0.688 |
| mm_Y1 | 0.6727 | 0.0828 | 0.5168 | 0.6701 | 0.8518 |
| mm_Y2 | 0.5447 | 0.0497 | 0.4502 | 0.5421 | 0.6484 |

**Table 3.** Posterior intervals and point estimates for correlation in random effects of the Hierarchical Bayesian Continuous Time Dynamic Models model of well-being and frequency of smartphone use. A graphic representations of correlations is reported in Figure 1.

| **Variable** | **Mean** | **Standard deviation** | **Confidence intervals** | | | **z < ±1.96** |
| --- | --- | --- | --- | --- | --- | --- |
|  |  |  | **2.50%** | **50%** | **97.50%** |  |
| drift_eta1__T0m_eta1 | 0.3748 | 0.154 | 0.045 | 0.3843 | 0.6408 | 2.4334 |
| mvarY1__T0m_eta1 | 0.2643 | 0.1094 | 0.0402 | 0.266 | 0.467 | 2.4166 |
| mm_Y1__T0m_eta1 | -0.4882 | 0.1503 | -0.7325 | -0.5059 | -0.1574 | -3.2483 |
| drift_eta1__T0m_eta2 | 0.3222 | 0.1304 | 0.0542 | 0.325 | 0.5662 | 2.4704 |
| drift_eta1_eta2__drift_eta1 | -0.3751 | 0.1835 | -0.6836 | -0.3859 | 0.0147 | -2.0439 |
| diff_eta1__drift_eta1 | -0.4519 | 0.1151 | -0.6505 | -0.4599 | -0.1979 | -3.925 |
| diff_eta2__drift_eta1 | 0.3531 | 0.137 | 0.0654 | 0.3671 | 0.5858 | 2.5768 |
| mvarY1__drift_eta1 | -0.361 | 0.1143 | -0.5684 | -0.3665 | -0.1327 | -3.1595 |
| mm_Y1__drift_eta1 | -0.4247 | 0.1322 | -0.6609 | -0.4379 | -0.1455 | -3.2118 |
| mm_Y2__drift_eta1 | 0.3486 | 0.1508 | 0.0435 | 0.3566 | 0.6187 | 2.3121 |
| diff_eta1__drift_eta1_eta2 | 0.3735 | 0.1568 | 0.0405 | 0.3874 | 0.6396 | 2.3816 |
| mm_Y2__drift_eta1_eta2 | -0.4983 | 0.1649 | -0.7541 | -0.525 | -0.1288 | -3.0223 |
| diff_eta1__drift_eta2_eta1 | 0.4193 | 0.1892 | -0.0022 | 0.4326 | 0.7335 | 2.2161 |
| mvarY1__drift_eta2_eta1 | 0.5422 | 0.1566 | 0.1813 | 0.564 | 0.7983 | 3.462 |
| mm_Y2__drift_eta2 | 0.3629 | 0.1726 | -0.0085 | 0.3778 | 0.6596 | 2.1023 |
| mvarY1__diff_eta1 | 0.6771 | 0.1439 | 0.336 | 0.7074 | 0.8654 | 4.7052 |
| mm_Y2__diff_eta2 | 0.6371 | 0.1198 | 0.3685 | 0.6562 | 0.8204 | 5.3175 |


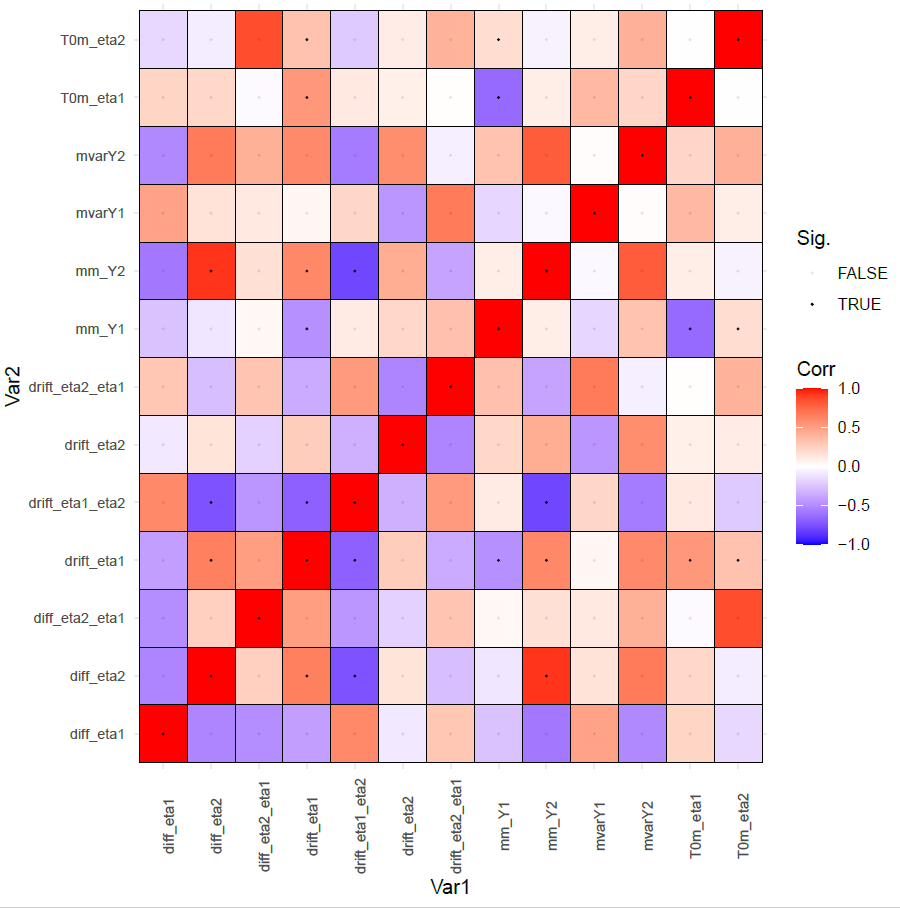
**Figure 1.** Graphic representation of correlations of Model 1.

**Table 4**. Population standard deviations and posterior intervals of the Hierarchical Bayesian Continuous Time Dynamic Models model of well-being and duration of smartphone use. This table shows how much individual parameters tend to differ from population mean values, i.e. the unexplained between-person variance (=heterogeneity of the effects)..

| **Variable** | **Mean** | **Standard deviation** | **Confidence intervals** | | |
| --- | --- | --- | --- | --- | --- |
|  |  |  | **2.50%** | **50%** | **97.50%** |
| T0m_eta1 | 0.7595 | 0.0714 | 0.6294 | 0.7575 | 0.9081 |
| T0m_eta2 | 0.6401 | 0.043 | 0.5586 | 0.6399 | 0.7263 |
| drift_eta1 | 0.884 | 0.1011 | 0.6813 | 0.88 | 1.0829 |
| drift_eta1_eta2 | 0.0535 | 0.0156 | 0.0283 | 0.0521 | 0.0877 |
| drift_eta2_eta1 | 0.2646 | 0.0545 | 0.1581 | 0.2639 | 0.3745 |
| drift_eta2 | 0.4496 | 0.0658 | 0.3262 | 0.4468 | 0.5807 |
| diff_eta1 | 3.4599 | 0.3406 | 2.845 | 3.442 | 4.1873 |
| diff_eta2_eta1 | 0.088 | 0.0327 | 0.0287 | 0.0872 | 0.1529 |
| diff_eta2 | 2.1556 | 0.329 | 1.5351 | 2.1559 | 2.8285 |
| mvarY1 | 2.2919 | 0.4309 | 1.5055 | 2.27 | 3.2035 |
| mvarY2 | 0.1495 | 0.1839 | 0.0052 | 0.0728 | 0.6557 |
| mm_Y1 | 0.7038 | 0.0856 | 0.5466 | 0.6981 | 0.8768 |
| mm_Y2 | 0.5326 | 0.0453 | 0.4527 | 0.5319 | 0.6251 |

**Table 5.** Posterior intervals and point estimates for correlation in random effects of the Hierarchical Bayesian Continuous Time Dynamic Models model of well-being and duration of smartphone use. A graphic representation of correlations is reported in Figure 2.

| **Variable** | **Mean** | **Standard deviation** | **Confidence intervals** | | | **z < ±1.96** |
| --- | --- | --- | --- | --- | --- | --- |
|  |  |  | **2.50%** | **50%** | **97.50%** |  |
| drift_eta1__T0m_eta1 | 0.5702 | 0.1175 | 0.2937 | 0.5862 | 0.7546 | 4.8524 |
| drift_eta2_eta1__T0m_eta1 | -0.4322 | 0.1679 | -0.6996 | -0.4479 | -0.0764 | -2.5741 |
| mvarY1__T0m_eta1 | 0.3484 | 0.0964 | 0.1596 | 0.3558 | 0.5337 | 3.614 |
| mm_Y1__T0m_eta1 | -0.5489 | 0.0903 | -0.6963 | -0.5594 | -0.341 | -6.0788 |
| mm_Y2__T0m_eta1 | -0.3021 | 0.1025 | -0.5003 | -0.3091 | -0.0895 | -2.9476 |
| drift_eta1__T0m_eta2 | 0.5015 | 0.1144 | 0.2655 | 0.5086 | 0.7029 | 4.3852 |
| diff_eta1__T0m_eta2 | -0.2196 | 0.0993 | -0.3926 | -0.2283 | -0.0001 | -2.2124 |
| mvarY1__T0m_eta2 | 0.298 | 0.1032 | 0.0829 | 0.3064 | 0.484 | 2.8871 |
| mm_Y2__T0m_eta2 | -0.3417 | 0.1028 | -0.5326 | -0.3451 | -0.124 | -3.3255 |
| diff_eta1__drift_eta1 | -0.5519 | 0.0764 | -0.6841 | -0.5564 | -0.3908 | -7.2248 |
| mm_Y1__drift_eta1 | -0.2847 | 0.1169 | -0.4905 | -0.2933 | -0.0438 | -2.4356 |
| diff_eta2__drift_eta1_eta2 | 0.4494 | 0.13 | 0.1526 | 0.4622 | 0.6562 | 3.4583 |
| mm_Y1__drift_eta1_eta2 | -0.4305 | 0.2133 | -0.7431 | -0.4653 | 0.0396 | -2.0181 |
| mvarY1__drift_eta2_eta1 | 0.3599 | 0.1635 | -0.0043 | 0.3694 | 0.6425 | 2.2008 |
| diff_eta2__drift_eta2 | 0.2656 | 0.122 | 0.0099 | 0.276 | 0.4763 | 2.1766 |
| mm_Y2__drift_eta2 | 0.6072 | 0.1148 | 0.351 | 0.6212 | 0.7897 | 5.2893 |
| mm_Y2__diff_eta2 | 0.6129 | 0.1371 | 0.2972 | 0.6384 | 0.8112 | 4.4723 |


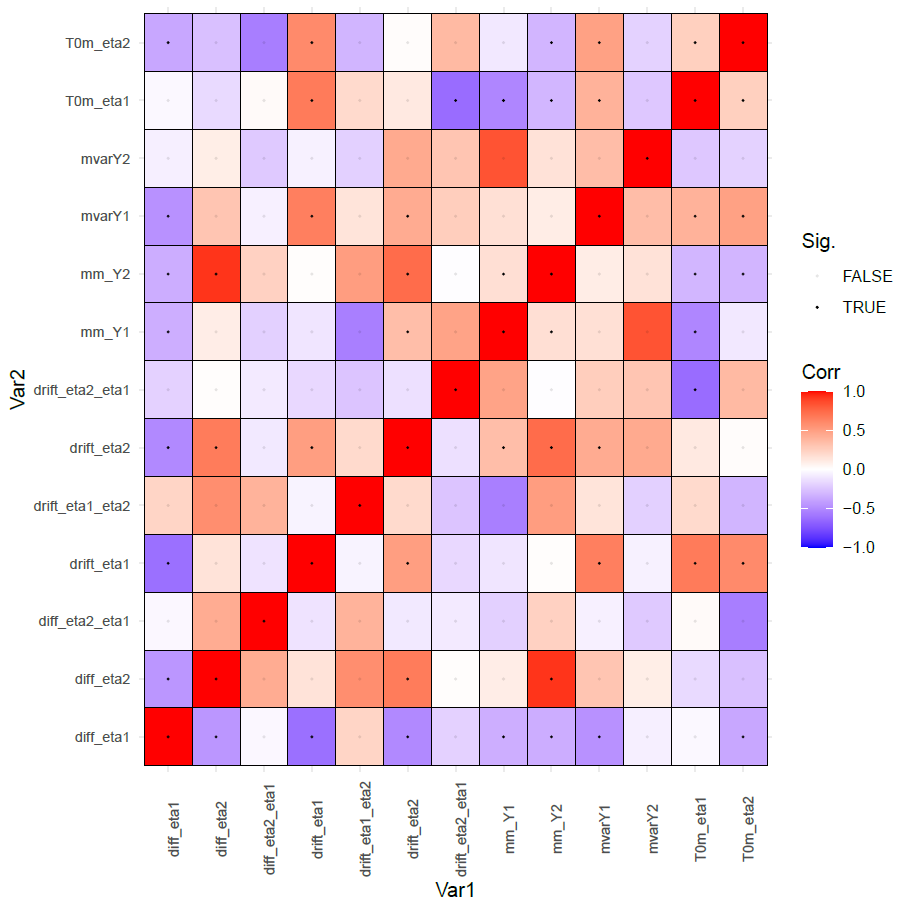


**Figure 2.** Graphic representation of correlations of Model 2.


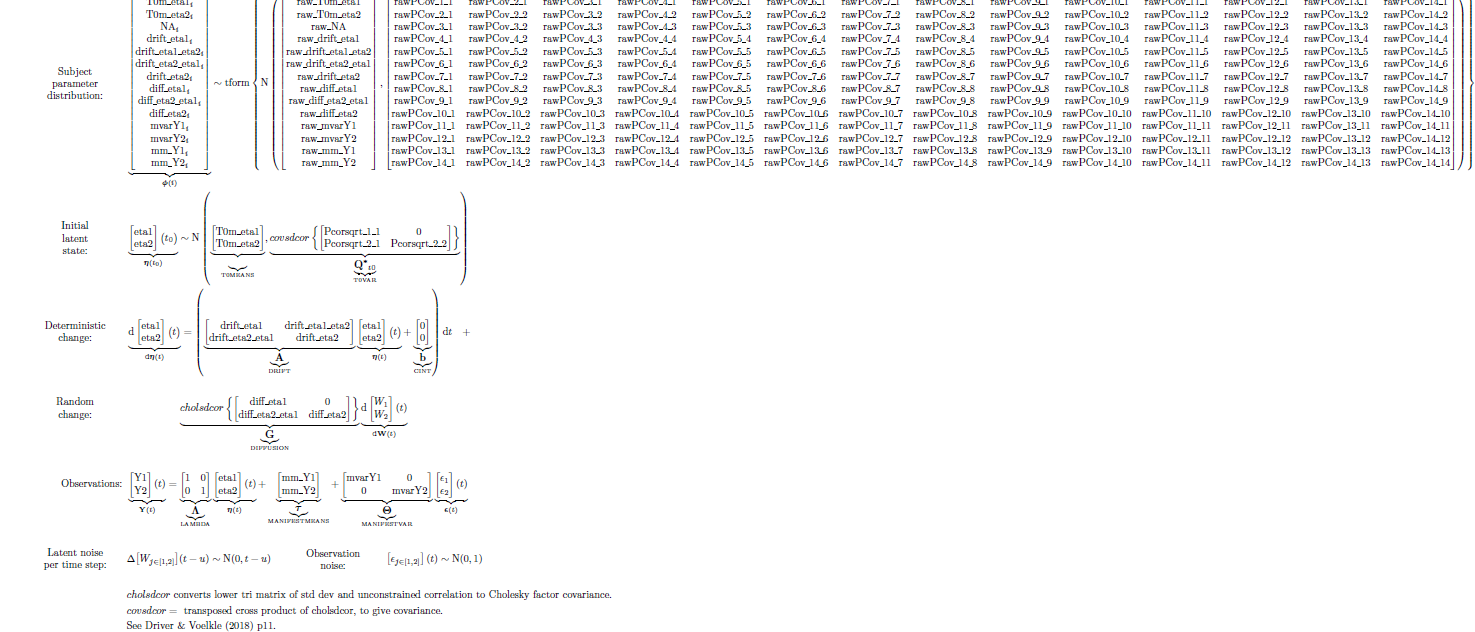


**Figure 3.** Model matrix.
